# Long-term follow-up of a healthy man with endogenous Streptococcus anginosus endophthalmitis

**DOI:** 10.1186/s12348-023-00383-w

**Published:** 2024-02-01

**Authors:** Juan Martin Sanchez, Mauricio Davila, Michael Halpert, Radgonde Amer

**Affiliations:** 1 Department of Ophthalmology, Hadassah Medical Center, Jerusalem, Israel; 2 Department of Ophthalmology, Hadassah University Hospital, POB 12000, 91120 Jerusalem, Israel

**Keywords:** Streptococcus anginosus, Endogenous endophthalmitis, Immunocompetent patient, Streptococcus milleri group

## Abstract

We report the long-term follow-up of an immunocompetent patient who presented with slowly progressive endogenous endophthalmitis secondary to *Streptococcus anginosus*. A 46-year-old healthy man presented with a two-month history of right eye iritis. On examination, visual acuity was 20/60 with intraocular pressure of 6 mm Hg. There was a small layer of hypopyon with non-granulomatous anterior uveitis and vitritis. On funduscopy, fluffy white peripheral retinal and pre-retinal lesions were noted in superonasal periphery. The patient denied any present or past illness. Diagnostic pars plana vitrectomy was performed. Culture and polymerase chain reaction of the vitreous sample were positive for *Streptococcus anginosus*. Intravitreal vancomycin and ceftazidime and systemic ceftriaxone were administered. Work-up which included blood and urine cultures, chest x-ray, echocardiography and abdominal ultrasound was unyielding. Subsequently and because of persistent post-infectious inflammatory reaction, intravitreal and oral steroids were administered in addition to oral azathioprine later on. After one year of follow-up, visual acuity was 20/20 with near vision of Jaeger 3 + and no signs of active uveitis were seen. Therefore, *Streptococcus anginosus* should be considered in the differential diagnosis of a slowly progressive endophthalmitis also in immunocompetent individuals.

## Introduction

Endogenous endophthalmitis (EE)is an intraocular infection caused by hematogenous spread of organisms from a non-ocular focus [1]. It is a major cause of severe visual morbidity and associated mortality rate [1]. It represents a diagnostic challenge in the early stages of the disease [1]. Systemic and topical antibiotics and vitrectomy are effective. However, the visual outcome is generally poor, and the infection may eventually lead to blindness [1–3]. The poor outcome is thought to be due to a delay in the diagnosis, virulence of the microorganisms, delay in eye surgery because of poor systemic condition and compromised wound healing after surgery [3].

We report on the long-term follow-up of a healthy man who presented with low grade uveitis that was subsequently disclosed to be endogenous bacterial endophthalmitis (EBE) secondary to *Streptococcus anginosus* (SA).

## Case report

A 46-year-old healthy male teacher, presented to the emergency room complaining of decreased visual acuity (VA) in the right eye (RE) of two-month duration. He had been previously treated in another outpatient clinic with topical steroids because of RE iritis. He was referred by the treating ophthalmologist because of non-responsive uveitis and the appearance of hypopyon.

**Fig. 1:**
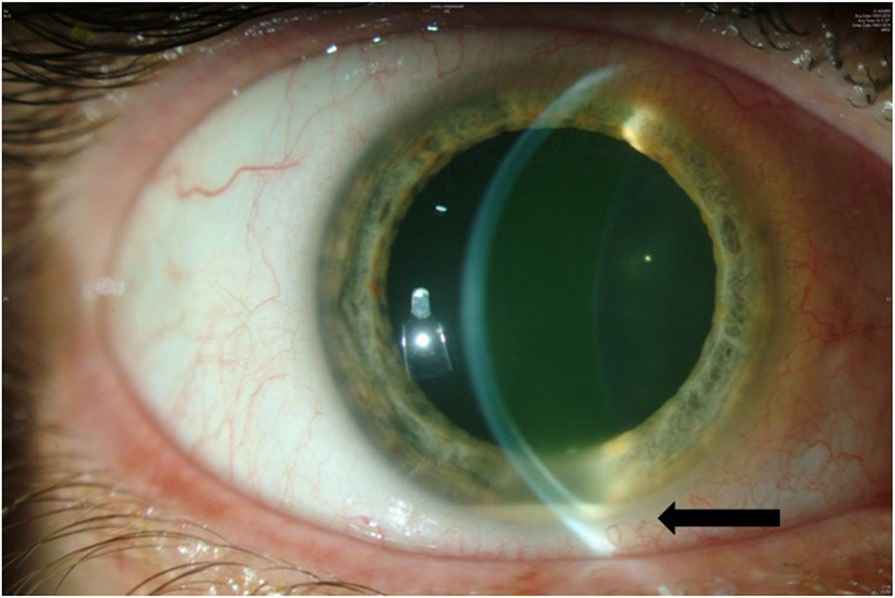
Anterior segment photograph of the right eye showing cold hypopyon (arrow) of less than 1 mm at presentation

**Fig. 2:**
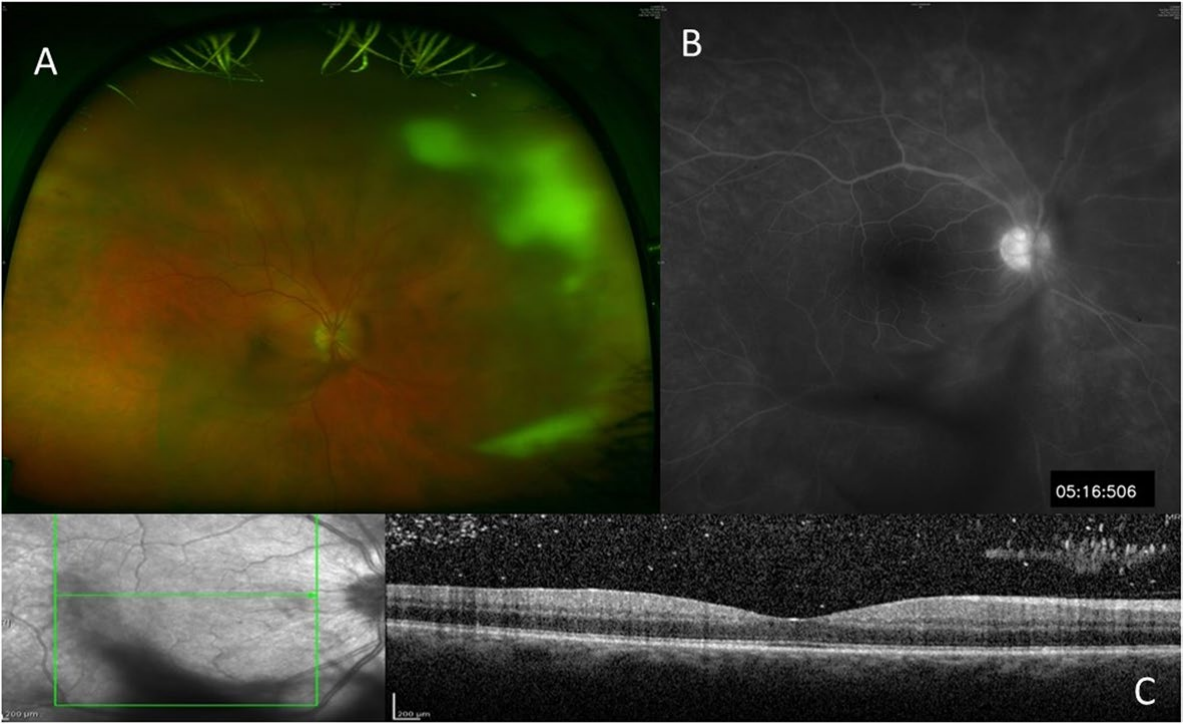
At presentation. **A** Wide-angle fundus photograph showing fluffy white preretinal and retinal lesions in the superonasal periphery and central vitreous opacity covering the posterior pole. **B** Fluorescein angiogram showing optic disc and fine capillary hyperfluorescence. **C** Optical coherence tomography showing preserved foveal contour and vitreous cells

On presentation, VA was RE 20/60, left eye (LE) VA was 20/20. LE exam was normal. In the RE, there was quiet conjunctiva, fine keratic precipitates in the inferior 2/3 of the cornea, anterior chamber cells + 3, flare + 2, hypopyon of less than 1 mm (Fig. 1) and vitritis (BIO Score + 1). Funduscopy revealed fluffy white elevated retinal and pre-retinal lesions in the superonasal periphery (Fig. 2A) and a small white retinal lesion inferiorly. Spectral-Domain optical coherence tomography (OCT) showed normal foveal contour (Fig. 2C). Fluorescein angiography showed a hot optic disc and fine capillary leakage (Fig. 2B). B-scan ultrasound showed dense point-like echoes in the vitreous cavity and a small elevated lesion superonasally. High frequency ultrasound did not reveal ciliary body abnormality.

Review of systems did not reveal an underlying past or present systemic condition or prior surgical intervention. The patient denied recent oropharyngeal or dental procedures, sinus or orbital conditions or use of intravenous drugs or immunosuppression of any kind. Work-up that included complete blood count, kidney and liver function test, erythrocyte sedimentation rate, C-reactive protein was unyielding. Because of the atypical course of uveitis and the appearance of cold hypopyon and fluffy white retinal lesions, the suspicion of EE was raised. The patient was hospitalized and underwent pars plana vitrectomy. A vitreous sample was sent for culture and polymerase chain reaction (PCR). Intravitreal vancomycin (1 mg/0.1 ml) and ceftazidime (2.25 mg/0.1 ml) were administered at the end of the operation.

There was a positive yield of SA in the vitreous cultures and real-time PCR confirmed the result. Additional work-up that included blood, urine cultures, chest X-ray, abdominal ultrasound, transesophageal echocardiography, serological tests for *Treponema pallidum, Brucella, Coxiella, Toxocara, Toxoplasma* and *Bartonella* was negative.

Treatment was initiated with intravenous (IV) ceftriaxone (2 g) twice per day for a period of six weeks. Prednisone (1 mg/day) was introduced one week later and it was tapered thereafter. Gradual regression of the chorioretinal lesions was noted and there was resolution of anterior uveitis and RE VA improved to 20/30 (near vision of Jaeger 2).

**Fig. 3:**
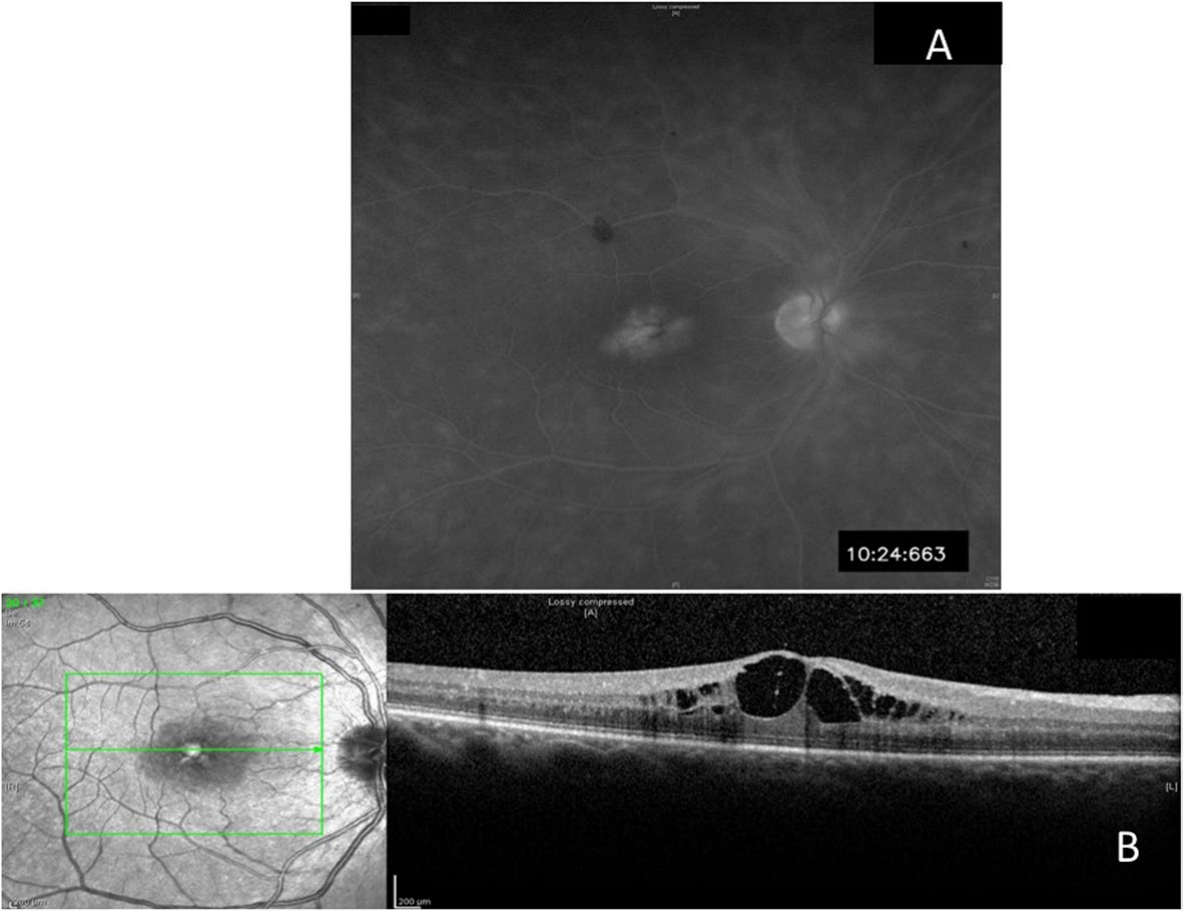
Three months after presentation. **A** Fluorescein angiogram (late phase) showing leakage from the optic disc and macular leakage. **B** Optical coherence tomography showing cystoid macular edema

Two months following the initial presentation, cystoid macular edema (CME) developed and reactivation of the chorioretinitis was suspected. A second course of IV ceftriaxone (2 g/day) was initiated for a period of 8 weeks in combination with prednisone. Because of persistent intraocular inflammation, refractory CME and optic disc neovascularization (NVD) (Fig. 3), a pulse of IV methylprednisolone (1gr/day) was administered over five days. NVD and CME resolved and the peripheral chorioretinal lesions became fibrotic. VA RE was 20/20. Subsequently, azathioprine was added (100 mg/day) to prednisone.

Because of resurgence of CME on prednisone taper, intravitreal triamcinolone acetonide (4 mg/0.1 ml) was administered (five months following the initial presentation). CME resolved completely and there was no recurrence thereafter.

**Fig. 4:**
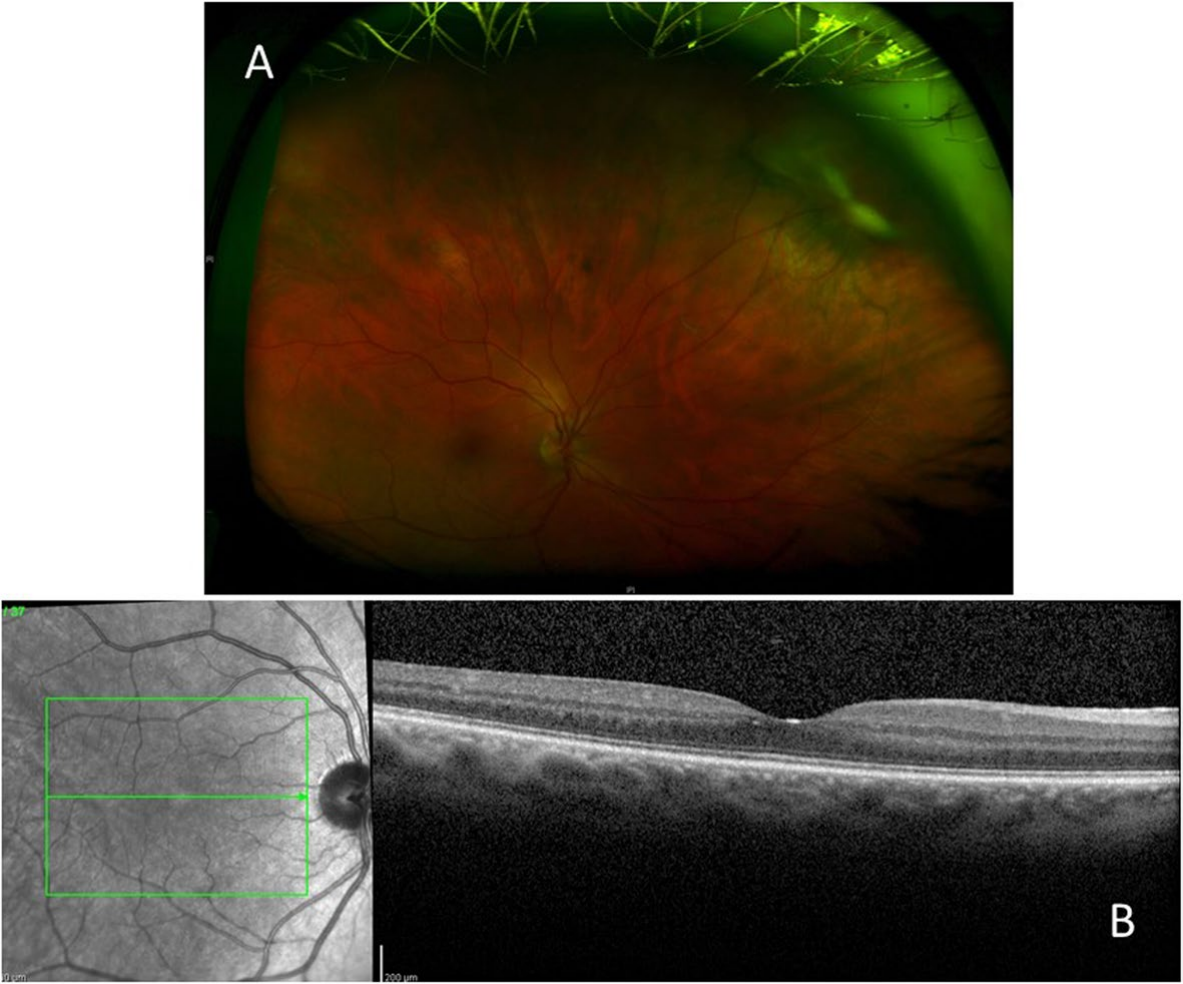
One year after presentation. **A** Wide-angle fundus photograph showing a fibrotic inactive chorioretinal scar in the superonasal peripheral retina. **B** Optical coherence tomography showing normal foveal contour and absence of vitreous cells

At one-year follow-up, RE VA remained 20/20 (near VA Jaeger 3) with no signs of active uveitis. The patient was maintained on prednisone (4 mg/day) and azathioprine (150 mg/day). OCT showed no CME (Fig. 4).

## Discussion

We herein report on the long-term follow-up of a healthy immunocompetent middle-aged patient who was diagnosed with EE secondary to *Streptococcus anginosus*. The patient presented with cold hypopyon and low-grade uveitis of two-month duration. It was an atypical presentation for a bacterial infection since bacterial endophthalmitis classically presents with an abrupt course and rapid deterioration. Fungal infections on the other hand present insidiously with slow progression [1]. Hypopyon, which is composed primarily of neutrophils, is seen in 35% [1] of EE of bacterial origin. It is termed as 'hot' in the presence of ciliary injection, and 'cold' in the absence of ciliary injection. Cold hypopyon is more commonly seen in patients with uveitis masquerade syndromes [4].

Despite the initial good response to ocular and systemic antibiotics and systemic steroids, persistent intraocular inflammation leading to CME and NVD necessitated aggressive immunosuppressive therapy with IV and intravitreal steroids in addition to azathioprine. When compared with the literature, visual prognosis in EE is usually poor. In a major review by Jackson et al. [1] on 342 cases of EBE, 41% of eyes achieved VA of ≥ 20/200, 35% had VA worse than < 20/200, and 19% required enucleation or evisceration in cases after 2001. Both intravitreal dexamethasone and vitrectomy were each associated with a significantly greater probability of retaining 20/200 vision or better and significantly fewer rates of evisceration or enucleation.

EE occurs when organisms reach the eye via the bloodstream, and then cross the blood ocular barrier. It is a rare but severe infection with poor visual prognosis and an appreciable mortality rate. EBE constitutes 2 to 6% of all cases of endophthalmitis [1]. Diagnostic challenge is encountered in the early stages of the disease, with 16% to 63% of the cases being initially misdiagnosed [1]. EE is frequently associated with many underlying systemic risk factors [1]. The most common risk factors include recent hospitalization, diabetes mellitus, urinary tract infection, immunosuppression, neutropenia, HIV, intravenous drug abuse and indwelling catheters [1].

*Streptococcus anginosus* group, also known as the *S. milleri* group (SMG), is a subgroup of *S. viridans* that consists of three distinct streptococcal species: *S. anginosus*, *S. intermedius*, and *S. constellatus* [5, 6]. SMG is found in the oral cavity, nasopharynx, throat, and sinuses. It was reported in association with infective endocarditis, sinusitis, orbital cellulitis, intraorbital abscess formation, and cavernous sinus thrombosis [5–7]. EBE resulting from SMG was rarely reported previously [2, 8–11].

Table 1summarizes 6 cases (5 males) who were diagnosed with SA-associated EE. The median age at diagnosis was 52 years. In three patients, EE was secondary to infective endocarditis (one also had liver abscess). In one patient it was secondary to liver abscess. Blood cultures were positive in all former 4 cases. The youngest reported patient, a 25-year-old woman was healthy with no underlying focus of infection and negative blood culture similar to the index case [9]. She presented with thrombocytosis and elevated CRP and indicated prior facial cellulitis that was treated with oral antibiotics. The index case however was that of a healthy man with no past or present history of any systemic illness or any prior surgical intervention. We speculate that EE in the index case resulted from a remote incidence of transient bacteremia in which bacteria disseminated in the bloodstream and colonized the retina with subsequent infection of the vitreous. Transient bacteremia classically lasts for minutes to a few hours before being cleared from the body, and it is typically harmless in healthy people [12]. This can occur after manipulation of organs normally colonized by bacteria, such as the oral mucosa during tooth brushing, flossing, or dental procedures, or instrumentation of the bladder or colon [12, 13].

**Table 1:** Demograhic characteristics, co-morbidities, primary focus of infection, presenting and final visual acuity, medical and surgical treatment, vitreous and blood cultures, follow-up period and systemic and ocular complications

	**Age/gender/ Eye**	**Co-morbidities**	**Primary focus of infection**	**Presenting VA** **Final VA**	**Medical therapy**	**Vitreous culture/Blood culture**	**Surgical intervention**	**Location of retinal infiltrate**	**Follow-up period**	**Systemic or Ocular complications**
Present Study, 2023	46/M/RE	None	None	20/60 20/20	IV ceftriaxone, P, IVI vancomycin, ceftazidime and TA	+/-	PPV	Superonasal and inferior peripheral retina	One year	CME, NVD
Lin et al., [9] 2023	25/F/LE	None	Remote history of facial cellulitis and possible sinusitis	CF HM	IVI voriconazole, clindamycin, and vancomycin (Empiric therapy)	+/-	PPV	Overlying macula and optic nerve	2months	Fibrotic changes of macular lesion, loss of foveal contour and outer retinal structures on OCT
Hadid et al., [2] 2005	62/M/LE	Recovered of NHL, poorly controlled NIDDM, anemia of chronic disease, recurrent oral candidiasis	SBE, mitral regurgitation with signs of vegetation and a probable tear in anterior mitral leaflet	20/200 HM	IV benzylpenicillin, gentamicin IVI vancomycin, ceftazidime	-/ +	PPV	Superonasal to optic disk	4weeks	Emergency mitral valve replacement for disruption of anterior mitral leaflet
Koay et al., [10] 2012	52/M/LE	None	Diverticular disease that likely predisposed to liver abscesses	CF 6/18	3-week course of IV amoxicillin, gentamicin, later on, 3-week course of oral amoxicillin	+ AC/ +	none	Superior temporal quadrant	3weeks	none
Itoh et al., [11] 2010	60/M/BE	Severe gingivitis	Brain abscess in left occipital lobe and infectious endocarditis	RE 0.02 0.7 LE 0.6 0.7	IV imipenem	-/ +	RE PPV, draining subretinal abscess and phacoemulsification with IOL implantation	RE dense vitritis obscuring fundus view, LE white mass on fovea	10months	Mitral valve replacement, and total dental extraction
Okada et al., [8] 1994	75/M/NR	NR	Endocarditis and liver abscess	Presenting VA-NR Final-HM	IVI antibiotics	+ / +	PPV	Panuveitis	NR	NR

*VA* Visual acuity, *M* male, *F* female, *RE* right eye, *LE* left eye, *BE* both eyes, *IV* intravenous, *IVI* intravitreal injection, *P* prednisone, *TA* triamcinolone acetonide, *PPV* pars plana vitrectomy, *NVD* optic disc neovascularization, *CME* cystoid macular edema, *CF* counting fingers, *HM* hand motion, *OCT* optical coherence tomography, *NHL* non- Hodgkin’s lymphoma, *NIDDM* non-insulin dependent diabetes mellitus, *SBE* subacute bacterial endocarditis, *AC* anterior chamber, *IOL* intraocular lens, *NR* not reported

The index case is unique given the lack of clinical or laboratory evidence of any systemic predisposition to infection and the excellent visual outcome after the longest follow-up of such a case in the literature.

In conclusion, SMG has predisposition to create pyogenic infections complicated by multiple abscesses. SA and the SMG group, known to be virulent organisms, should be contemplated in the differential diagnosis of a slowly progressive endophthalmitis even in immunocompetent patients. Improved outcome is associated with prompt surgical and medical intervention including local and systemic antimicrobial treatment in combination with anti-inflammatory therapy.

## Data Availability

No datasets were generated or analysed during the current study.
